# Impact of chronic smoking on traumatic brain microvascular injury: An in vitro study

**DOI:** 10.1111/jcmm.16741

**Published:** 2021-06-23

**Authors:** Farzane Sivandzade, Faleh Alqahtani, Luca Cucullo

**Affiliations:** ^1^ Department of Biological Sciences Oakland University Rochester MI USA; ^2^ Department of Foundation Medical Studies Oakland University William Beaumont School of Medicine Rochester MI USA; ^3^ Department of Pharmacology and Toxicology College of Pharmacy King Saud University Riyadh Saudi Arabia

**Keywords:** alternative, blood‐brain barrier, endothelium, in vitro, inflammation, Nrf2, oxidative stress, smoking, TBI, traumatic injury

## Abstract

Traumatic brain injury (TBI) is a major reason of cerebrovascular and neurological damage. Premorbid conditions such as tobacco smoking (TS) can worsen post‐TBI injuries by promoting vascular endothelial impairments. Indeed, TS‐induced oxidative stress (OS) and inflammation can hamper the blood‐brain barrier (BBB) endothelium. This study evaluated the subsequence of chronic TS exposure on BBB endothelial cells in an established in vitro model of traumatic cell injury. Experiments were conducted on confluent TS‐exposed mouse brain microvascular endothelial cells (mBMEC‐P5) following scratch injury. The expression of BBB integrity–associated tight junction (TJ) proteins was assessed by immunofluorescence imaging (IF), Western blotting (WB) and quantitative RT‐PCR. We evaluated reactive oxygen species (ROS) generation, the nuclear factor 2–related (Nrf2) with its downstream effectors and several inflammatory markers. Thrombomodulin expression was used to assess the endothelial haemostatic response to injury and TS exposure. Our results show that TS significantly decreased Nrf2, thrombomodulin and TJ expression in the BBB endothelium injury models while increased OS and inflammation compared to parallel TS‐free cultures. These data suggest that chronic TS exposure exacerbates traumatic endothelial injury and abrogates the protective antioxidative cell responses. The downstream effect was a more significant decline of BBB endothelial viability, which could aggravate subsequent neurological impairments.

**Highlights**

- Chronic TS exposure exacerbates TBI‐induced inflammatory vascular responses, intracellular ROS generation, OS and blood haemostasis dysregulation.
- TS exposure inhibits the TBI‐activate antioxidative response system (ARS) by promoting the down‐regulation of Nrf2 and its downstream detoxifying effector molecules.
- Chronic TS exposure significantly increases the negative impact of TBI on BBB tight junctions' expression and distribution.
- Chronic TS exposure inducing BBB impairment and vascular inflammatory responses could substantially impact the physiological anti‐inflammatory and antioxidative responses to TBI, contributing to the significant exacerbation of post‐TBI neurovascular and neurological disorders.

## 1 INTRODUCTION

Traumatic brain injury (TBI) has been among the principal cause of disability and death for a long time, especially among the youth of industrialized societies.^1^, ^2^ As the Centers for Disease Control and Prevention (CDC) reports, annually, almost two and a half million people in the United States suffer from TBI following assaults, motor vehicle accidents, struck by objects, falls, incidents relevant to sports activities, etc. Furthermore, more than five million people in the United States live with a TBI‐caused lifelong disability.^3^, ^4^, ^5^ The pathophysiological mechanisms of TBI unfold into two sequential and interconnected phases, primary and secondary injury.^6^ Primary brain injury is an irreversible and major pathological factor. In contrast, secondary brain damage resulting from a physiological response to the initial traumatic injury is characterized by anatomical, molecular, cellular and behavioural alternations.^7^, ^8^, ^9^

Delayed secondary metabolic and biochemical alternations are precursory to other pathological processes at the cellular and molecular levels. These include oxidative stress (OS), inflammation, excitotoxicity, augmented vascular permeability, blood‐brain barrier (BBB) disruption, necrosis, apoptosis and mitochondrial dysfunction. The downstream effect worsened post‐TBI and subsequent neuronal dysfunction.^1^, ^9^, ^10^ Inflammation is a crucial contributor to the TBI pathophysiology, characterized by peripheral inflammation, cell infiltration and inflammatory mediators' secretion.^11^, ^12^ Preserved and extra‐inflammation cause exacerbation of subsequent neurological damage by secreting inflammatory cytokines, including IL‐6, TNF‐α and IL‐10. Their release is regulated via a signalling pathway controlled by nuclear factor kappa B (NF‐kB).^12^, ^13^

Blood‐brain barrier is pivotally involved in sustaining brain homeostasis, preventing potentially harmful substances from entering the central nervous system (CNS) and prohibiting the body's peripheral immune cells from penetrating the brain tissue. Hence, the brain may temporarily be exposed to the exogenous and endogenous elements, potentially hazardous for neuronal activities’ disruption of BBB integrity.^14^ Post‐traumatic impairment of the BBB is a severe factor resulting in the loss of nervous tissue and affecting neuroprotective drugs' response.^15^ Oxidative stress (OS) has been shown to promote TJ proteins' disruption (including ZO‐1, occludin and claudins) and impair BBB integrity.^16^ OS, which results from the uncontrolled ROS generation in the brain tissue, has been acknowledged as one of the main pathogenic factors for secondary injury post‐TBI.^17^, ^18^ Moreover, OS (and elevated intracellular levels of ROS) promotes the endothelial up‐regulation of cell adhesion molecules, increases the inflow of inflammatory cells into the CNS and releases pro‐inflammatory cytokines.

From this perspective, TS contains high levels of ROS and other toxic and carcinogenic compounds, damaging tissues and organs. Indeed, chronic TS exposure promotes BBB dysfunction by activating inflammatory, oxidative and immune responses, which assist the onset and/or progression of various neurological disorders, including TBI.^4^, ^19^ Therefore, it is unsurprising that chronic smoking influences the extent of TBI and post‐TBI recovery.^4^ The pro‐inflammatory tobacco activity may explain why TBI patients who are also chronic smokers present increased post‐traumatic cerebrovascular inflammation than non‐smokers.^5^ Nicotine is recognized as a leading cause of abnormal differentiation of neurons, decreased synaptic activity, promoted apoptosis and enhanced expression of several inflammatory adhesion molecules and cytokines causing abnormal brain development.^5^ Moreover, the nicotine in cigarettes becomes a vital factor in nicotine receptor activation, liberating nitric oxide from the vascular endothelium, thus increasing BBB permeability and hampering brain homeostasis.

It is well known that chronic TS promotes vascular endothelial dysfunction and increases the possibility of onset and progression of cerebrovascular diseases like ischaemic vessel disease, vascular dementia, stroke, Alzheimer's and multiple sclerosis. Recent studies strongly suggest that TS‐induced inactivation of protective response pathways can worsen TBI aftereffect and impact patients' recovery. However, the underlying mechanisms and characterization of shared critical mediators of BBB impairment caused by TBI and TS have not been thoroughly evaluated. Thus, this study's primary objectives were to assess the pathogenic factors through which chronic TS exposure can exacerbate the impact of traumatic injuries at the BBB endothelium. For this purpose, we investigated the effect of premorbid TS exposure on post‐traumatic microvascular damage using an in vitro BBB endothelial traumatic injury model generated from mBMEC‐P5 cells.

## 2 METHODS

### 2.1 Reagents and materials

Primary brain microvascular endothelial cells from C57 black 6 mice (C57BL/6‐mBMEC, #C57‐ 6023) and complete mouse endothelial cell medium (M1168) were acquired from Cell Biologics. Quantikine ELISA kits were obtained from R & D systems. Subcellular Protein Fractionation and Pierce BCA Protein Assay Kits (respectively #78840 and #23225) were purchased from Thermo Scientific. anti‐claudin‐5 (#352500) and rabbit anti‐Nrf2 (#PA5‐88084) were purchased from Invitrogen; the rest of the antibodies were purchased from other sources mentioned in our previous publications.^5^, ^20^

### 2.2 Cell culture

mBMEC cells were seeded on 6‐well plates containing fresh supplemented medium with 10% FBS for a final total volume of 2 mL/well. All cell cultures were maintained in an incubator at 37°C, 95% humidity and 5% CO_2_.^21^ Culture media was changed every 48 hours until the cells reached full confluency.

### 2.3 Soluble cigarette smoke extract preparation

Standardized 3R4F research cigarettes obtained from the National Institute on Drug abuse (NIDA) were used to prepare the soluble TS extract as previously described by our group using a computer‐controlled Single Cigarette Smoking Machine (SCSM) acquired from CH Technologies Inc. (35 mL puff volume, 2 seconds puff duration, 58 seconds intervals, 8 puffs per cigarette directly into phosphate‐buffered saline (PBS), equivalent to full flavour brands containing 9.4 mg tar and 0.726 mg nicotine/cigarette).^20^, ^21^, ^22^ For each exposure cycle, freshly prepared TS extract was diluted to 5% (V/V) concentrations in a culture medium containing 1% FBS.

### 2.4 Induction of traumatic endothelial injury in vitro

Confluent mBMEC‐P5 cultures were manually scratched as previously described in several published studies.^1^, ^23^, ^24^ In brief, every 6‐well plate was scratched manually using a sterile plastic pipette tip matching a 9 × 9 square grid with an inter‐line space of 4 mm. Without changing the media, cell cultures were stored back in the incubator for another 24 hours and maintained at 37℃ under normoxic conditions. Parallel uninduced cultures were prepared as controls.

### 2.5 Preparation of protein extracts and Western Blotting (WB)

We harvested total proteins by RIPA lysis buffer based on the manufacturer's guidelines to determine the protein expression levels. Total proteins were centrifuged at 14 000 *g* for 30 minutes. Protein samples were then divided into three groups based on their putative molecular weights. Specifically, Group 1 was used to detect the expression levels of ZO‐1, Nrf2 and HO‐1; Group 2: was used to detect Pecam‐1, NF‐kB and claudin‐5; and Group 3 was used to detect the expression levels of Vcam‐1, occludin and NQO‐1. Protein quantification was then assessed using a Pierce BCA protein assay kit. Samples containing 30 μg were then resolved on SDS‐PAGE, transferred to nitrocellulose membranes and then incubated overnight at 4℃ with the primary antibodies of interest.^21^, ^25^ The membranes were then washed and re‐incubated for 2 hours at 25℃ with the corresponding secondary antibodies for immunodetection.

### 2.6 Enzyme‐Linked Immunosorbent assay (ELISA)

The analysis of cell culture supernatant was done using Quantikine ELISA kits to determine the levels of thrombomodulin and inflammatory cytokines IL‐6, TNF‐α and IL‐10, as described by the manufacturer.

### 2.7 RNA extraction and quantitative Real‐Time Polymerase Chain Reaction (RT‐PCR)

Quantitative RT‐PCR was performed according to the protocol used in our previous work.^26^, ^27^ Briefly, the total RNA was extracted from the mBMEC cells using an RNeasy plus mini kit as described in the manufacture's protocol (Qiagen Inc.). Complementary DNA (cDNA) was synthesized using a superscript III first‐strand synthesis system (Life Technologies). Gene expression was determined by qPCR using an SYBR green‐based fluorescence procedure.^28^ The primer pairs (see sequences in Table 1) were designed based on PubMed GenBank and synthesized by Integrative DNA technologies. The RNA targets were amplificated using a Bio‐Rad CFX96 Touch Real‐Time PCR detection system.

**TABLE 1:** Forward and reverse primer sequences (5′‐3′) used in quantitative RT‐PCR

Target gene	Forward	Reverse
NRF2	5′‐ GGC TCA GCA CCT TGT ATC TT ‐3′	5′‐ CAC ATT GCC ATC TCT GGT TTG ‐3′
NQO‐1	5′‐ GAG AAG AGC CCT GAT TGT ACT G ‐3′	5′‐ ACC TCC CAT CCT CTC TTC TT ‐3′
HO‐1	5′‐ CTC CCT GTG TTT CCT TTC TCT C ‐3′	5′‐ GCT GCT GGT TTC AAA GTT CAG ‐3′
NF‐kB	5′‐ AGA CAT CCT TCC GCA AAC TC ‐3′	5′‐ TAG GTC CTT CCT GCC CAT AA ‐3′
Claudin‐5	5′‐ GGT GAA GTA GGC ACC AAA CT ‐3′	5′‐ TTT CTC CAG CTG CCC TTT C ‐3′
Occludin	5′‐ CAG CAG CAA TGG TAA CCT AGA G ‐3′	5′‐ CAC CTG TCG TGT AGT CTG TTT C ‐3′
VCAM‐1	5′‐ GAG GGA GAC ACC GTC ATT ATC ‐3′	5′‐ CGA GCC ATC CAC AGA CTT TA ‐3′
PECAM‐1	5′‐ CAA CAG AGC CAG CAG TAT GA ‐3′	5′‐ TGA CAA CCA CCG CAA TGA ‐3′
ZO‐1	5′‐ CAT TAC GAC CCT GAA GAG GAT G ‐3′	5′‐ AGC AGG AAG ATG TGC AGA AG ‐3′
Β‐Actin	5′‐ GAG GTA TCC TGA CCC TGA AGT A ‐3′	5′‐ CAC ACG CAG CTC ATT GTA GA ‐3′

### 2.8 Immunofluorescence imaging (IF)

Immunofluorescence imaging was performed according to a validated protocol used in our previous work.^26^, ^27^ mBMEC cells cultured in double‐well chamber slides were fixed in 4% formaldehyde (methanol‐free) and permeabilized at 25℃ by 0.02% Triton X‐100 for 10 minutes. The slides were then set in blocking buffer at 25℃ for 1 hours and then incubated with primary antibodies at 4℃ overnight. The following day, the slides were washed and then stained with either Alexa Fluor R 488–conjugated or Alexa Fluor R 555–conjugated goat antibodies at 25℃ and mounted with DAPI mounting medium to show the locations of the nuclei. Slides were then photographed using our EVOS M5000 Imaging System (Thermo Fisher, #AMF5000) following previously published procedures.^21^, ^25^

### 2.9 Cell viability assay

Tetrazolium 3‐(4, 5‐dimethyl thiazolyl‐2)−2, 5‐diphenyltetrazolium bromide (MTT) assay was used to assess the cells’ viability.^21^ Briefly, 24 hours after TBI induction, 5 mg/mL of MTT was added to the cells seeded on 6‐well plates and incubated for 3 hours at 37℃. Metabolically active cells converted the yellow MTT to purple formazan crystals. The formazan compound was solubilized using 1ml of DMSO, and the absorbance was then measured using Bio‐Rad plate reader at 570 nm.

### 2.10 Measurement of intracellular ROS generation

Intracellular ROS was analysed using 2,7‐dichlorodihydrofluorescein diacetate (2,7‐DCFH‐DA).^29^, ^30^ Briefly, cultured cells on 6‐well plates were rinsed with cold PBS and incubated in 25 umol/L DCFH‐DA for 45 minutes at 37℃ in the dark. Cells were then rinsed thrice with PBS and transferred to a 96‐well black plate by scraping the wells’ surface. The intensity of dichlorofluorescein (DCF) fluorescence was measured with a Bio‐Rad plate reader at specific excitation (Ex: 485 nm) and emission wavelengths (Em: 535 nm).

### 2.11 Glutathione level measurement

The analysis of cell lysate was performed using Quantification Kit for Oxidized and Reduced Glutathione (Sigma‐Aldrich). Specifically, we assessed the oxidized glutathione (GSSG) levels and reduced glutathione (GSH) according to the manufacturer's instruction. Fluorescence intensity was measured via a Bio‐Rad plate reader at specific excitation (Ex: 490 nm) and emission wavelengths (Em: 520 nm).

### 2.12 Statistical analysis

All the results were reported as mean values ± standard deviation (SD). GraphPad Prism 9 Software Inc. was used to perform a one‐way analysis of variance (ANOVA) followed by Tukey's or Dunnett's test to evaluate the significance of the data. *P* values ≤ .05 were considered statistically significant.

## 3 RESULTS

### 3.1 Evaluation of cell viability through MTT cytotoxicity assay

**FIGURE 1:**
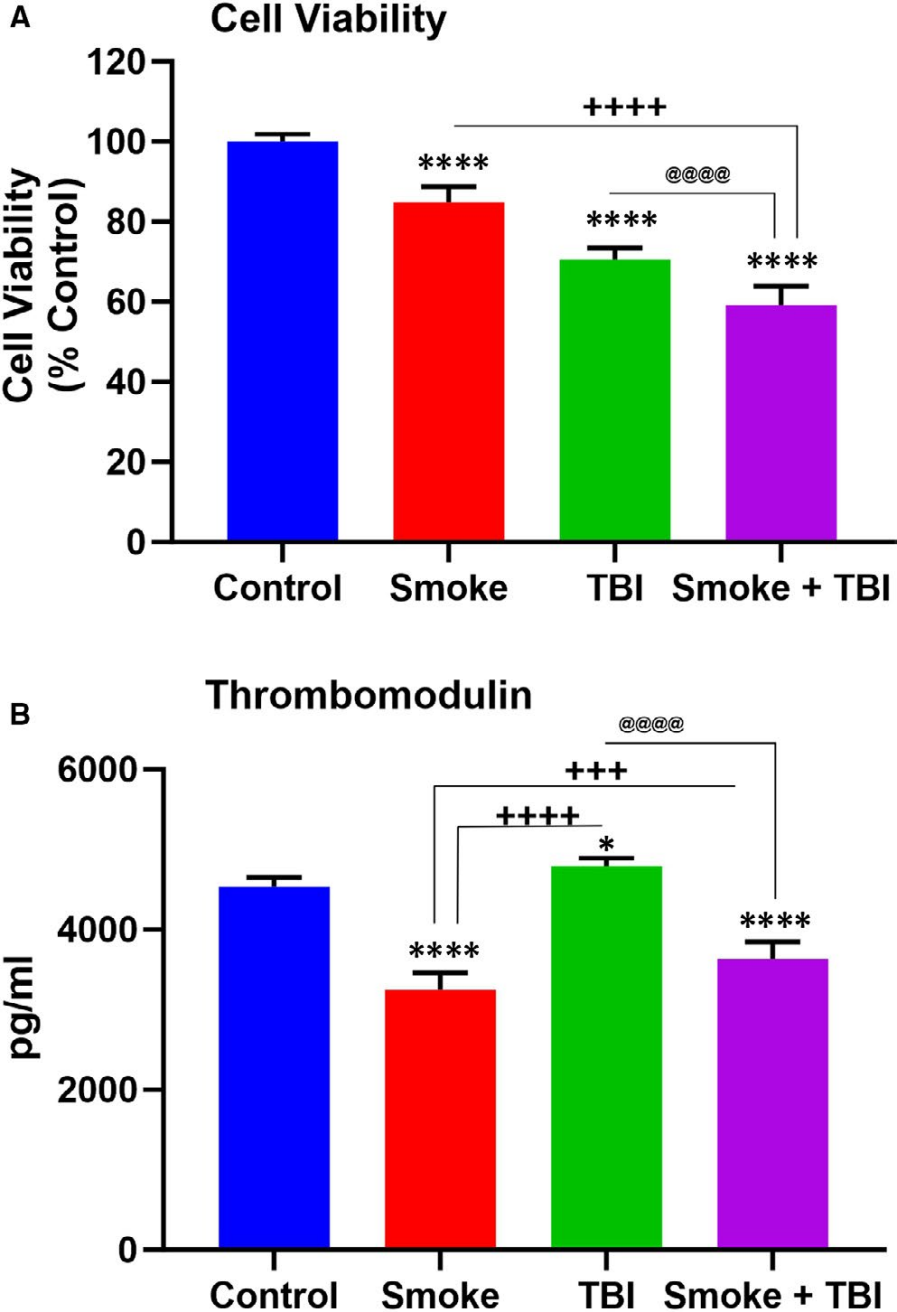
A, MTT cytotoxicity assay for cell viability evaluation. B, Effect of TS exposure and TBI on level of thrombomodulin. Levels of thrombomodulin in the supernatants collected at 24 h after TBI was measured by ELISA. TS exposure promoted suppression of thrombomodulin and potentially impaired blood haemostasis. n = 6 biological replicates, *P < .05, **P < .01, ***P < .001, ****P < .0001 vs control. ^+^ *P < *.05, ^++^ *P < *.01, ^+++^ *P < *.001, ^++++^ *P < *.0001 vs smoked group. ^@^ *P < *.05, ^@@^ *P < *.01, ^@@@^ *P < *.001, ^@@@@^ *P < *.0001 vs TBI‐induced group

MTT cytotoxicity assay was performed to evaluate TS and TBI's effect based on cell toxicity and viability. As indicated in Figure 1A, the viability of TS‐exposed and TBI‐induced cells significantly decreased compared to control.

### 3.2 TS Exposure increases the inflammatory endothelial responses to traumatic injury while possibly affecting blood haemostasis

Chronic TS exposure reduced the anticoagulant factor thrombomodulin expression level as previously recognized both in vitro and in vivo studies.^21^, ^31^ As shown in Figure 1B, a traumatic injury had an opposite impact on the cell cultures, which responded by up‐regulating thrombomodulin up‐regulation. As observed from the result, the TBI effect on the BBB endothelial cells was abolished by TS. These data suggest that TS can abolish the endothelial response to traumatic injuries and increase blood coagulation risk in TBI patients.

We used a combination of WB, IF and quantitative RT‐PCR to assess changes in the expression levels of NF‐kB (an essential modulator and inducer of inflammatory activities), inflammatory adhesion molecules, platelet endothelial cell adhesion molecule 1 (PECAM‐1) and vascular cell adhesion protein 1 (VCAM‐1).

**FIGURE 2:**
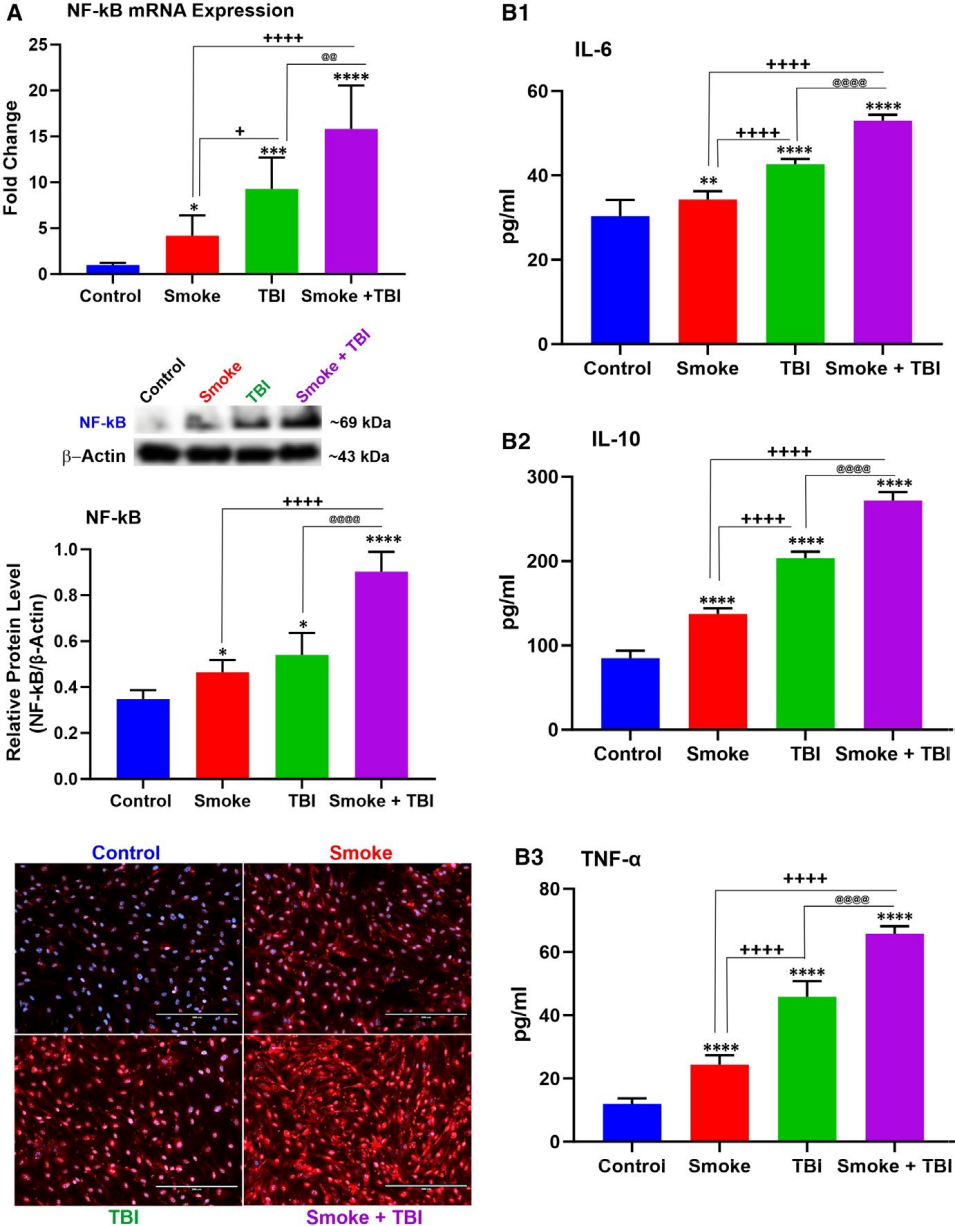
TS exposure elicits a stronger inflammatory response at the BBB endothelium following traumatic injury. A, NF‐kB was independently up‐regulated by TS exposure and traumatic injury. Also note the combined effect of TS over traumatic injury which potentiated the inflammatory response. Results were assessed by WB, quantitative RT‐PCR and immunofluorescence imaging. A similar trend was observed in respect to the expression levels of inflammatory cytokines (B1) IL‐6, (B2) IL‐10 and (B3) TNF‐α. 24 h after traumatic injury. n = 6 biological replicates, *P < .05, **P < .01, ***P < .001, ****P < .0001 vs control. ^+^ *P < *.05, ^++^ *P < *.01, ^+++^ *P < *.001, ^++++^ *P < *.0001 vs smoked group. ^@^ *P < *.05, ^@@^ *P < *.01, ^@@@^ *P < *.001, ^@@@@^ *P < *.0001 vs TBI‐induced group. WB analyses express protein/β‐actin ratios

**FIGURE 3:**
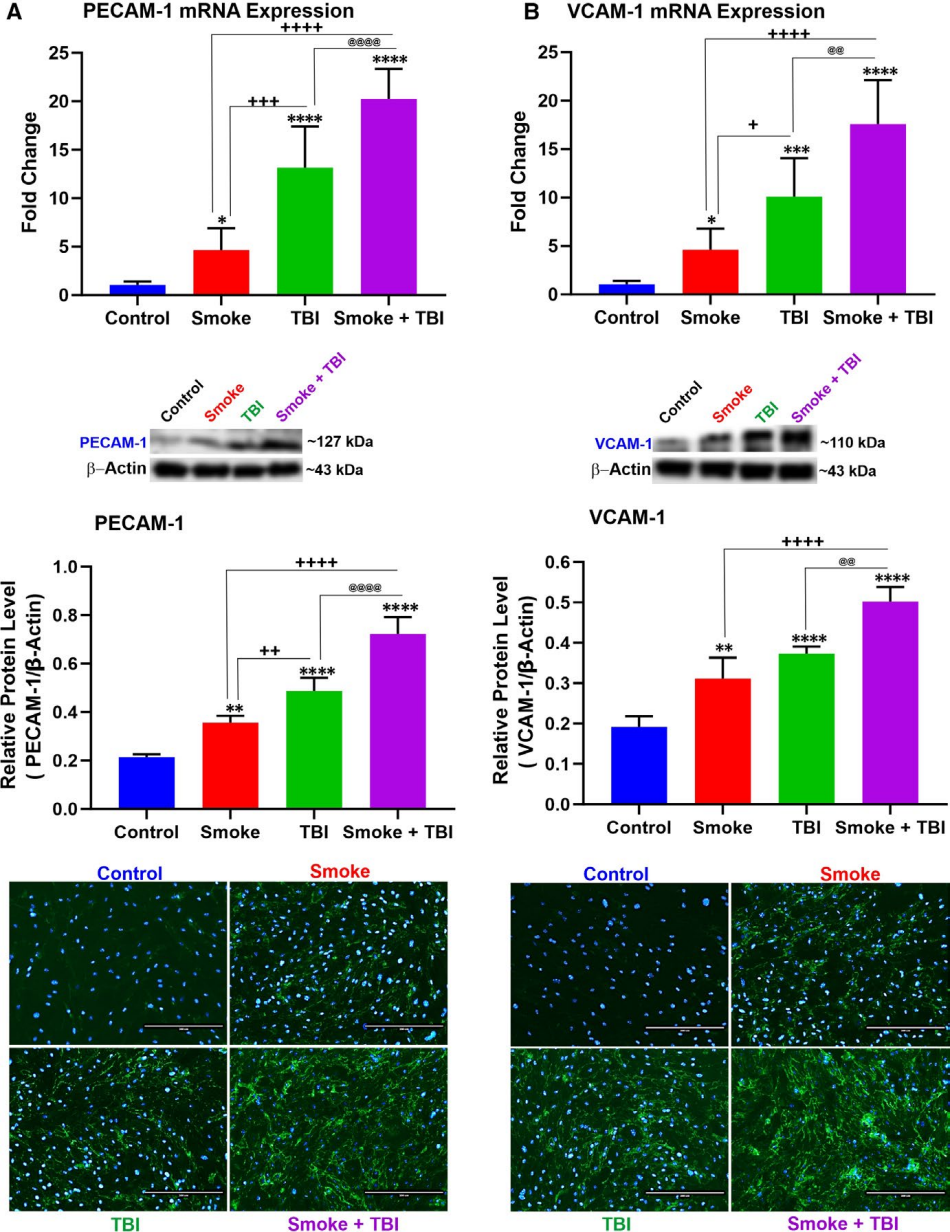
TS exposure enhances the expression levels of inflammatory adhesion molecules PECAM‐1 and VCAM‐1 in BBB endothelial cells undergoing traumatic injury simulation. TS exposure caused up‐regulation of PECAM‐1 expression and further enhanced by traumatic injury as shown by (A) WB, quantitative RT‐PCR and immunofluorescence analyses. B, A similar parallel trend was observed for VCAM‐1. Please note that the reported β‐actin band for PECAM‐1 (Figure 3A) is the same as that shown for Cld‐5 (Figure 6B) since these proteins were run in the same group (Group 2). Similarly, the reported β‐actin band for VECAM‐1 (Figure 3B) is the same as that shown for NQO‐1 (Figure 5C) and Ocln (Figure 6C) since these proteins were also run in the same group (Group 3) as stated in the method section. n = 6 biological replicates, *P < .05, **P < .01, ***P < .001, ****P < .0001 vs control. ^+^ *P < *.05, ^++^ *P < *.01, ^+++^ *P < *.001, ^++++^ *P < *.0001 vs smoked group. ^@^ *P < *.05, ^@@^ *P < *.01, ^@@@^ *P < *.001, ^@@@@^ *P < *.0001 vs TBI‐induced group. WB analyses express protein/β‐actin ratios

As shown in Figure 2A, we determined a remarkable NF‐kB increase (protein expression and mRNA levels). In parallel, we also observed an increased expression of inflammatory cytokines, including IL‐6 (Figure 2B), IL‐10 (Figure 2C) and TNF‐α (Figure 2D). A similar trend was also noted when we assessed the expression levels of PECAM‐1 (Figure 3A) and VCAM‐1 (Figure 3B). Both TS and TBI as standalone stimuli can elicit an inflammatory response. However, this response was substantially enhanced in cell cultures exposed to co‐stimulation (TBI +TS). These data suggest that TS exposure increases the vascular inflammatory response to traumatic injury.

### 3.3 TS Exposure increases ROS generation and OS

**FIGURE 4:**
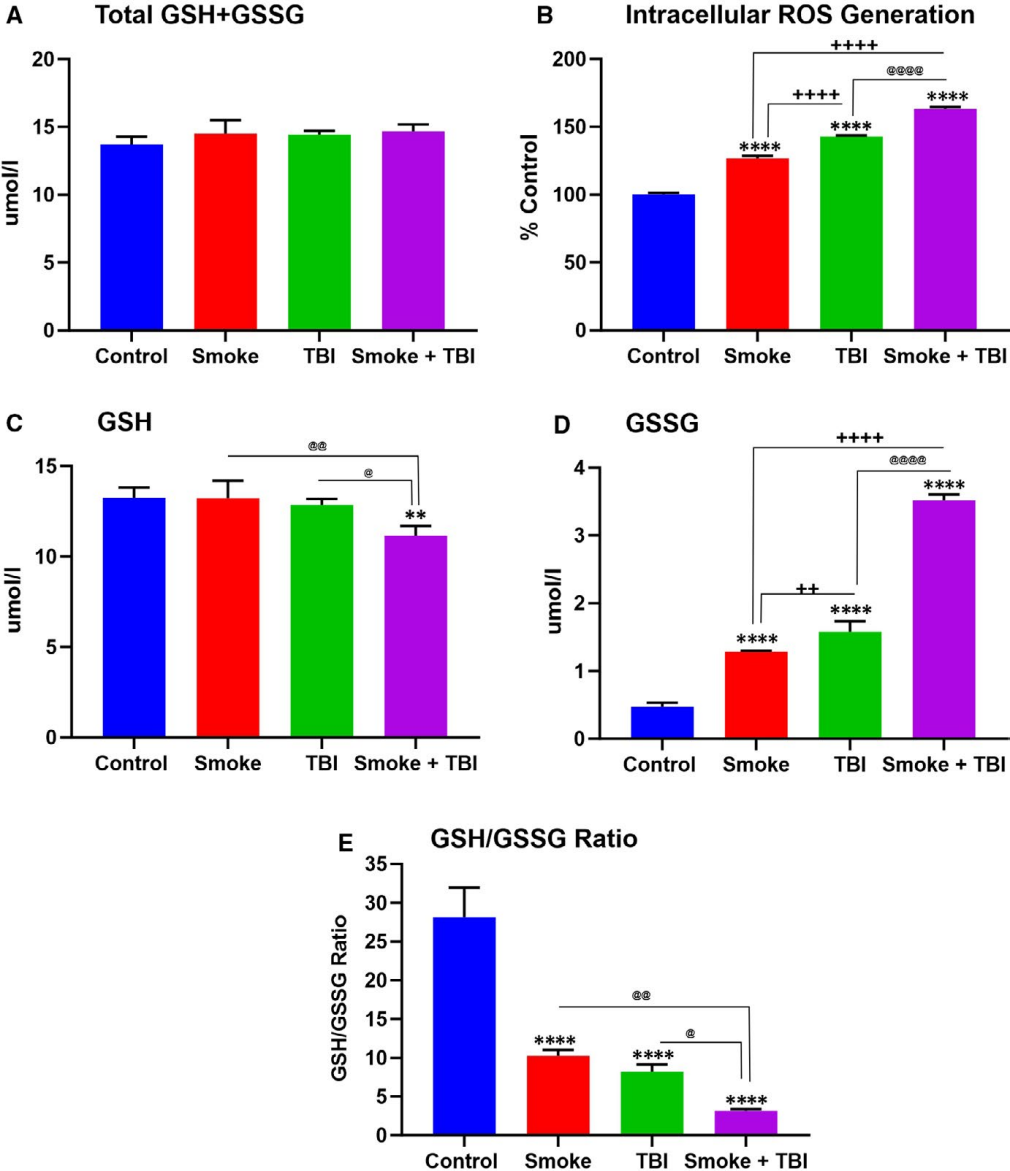
TS exposure and TBI affected intracellular ROS generation and oxidative stress. A, Total glutathione (GSH+GSSG), (B) intracellular ROS generation, (C) reduced glutathione (GSH), (D) oxidized glutathione (GSSG) and (E) GSH/GSSG measured by plate reader. n = 6 biological replicates, *P < .05, **P < .01, ***P < .001, ****P < .0001 vs control. ^+^ *P < *.05, ^++^ *P < *.01, ^+++^ *P < *.001, ^++++^ *P < *.0001 vs smoked group. ^@^ *P < *.05, ^@@^ *P < *.01, ^@@@^ *P < *.001, ^@@@@^ *P < *.0001 vs TBI‐induced group

The intracellular ROS generation and glutathione levels were assessed to determine any possible relation between oxidative stress and cell‐deteriorating effect. Measurements of total glutathione as GSH and GSSG (total GSH +GSSG) were not different from controls across all the experimental settings (see Figure 4A). However, as shown in Figure 4B, intracellular ROS was significantly higher in TS‐exposed samples as well as in TBI‐induced cultures. When TS pre‐exposure was combined with traumatic injury, ROS generation reached the highest level measured across all the experimental settings. The effect was statistically significant when compared to controls as well as TS and TBI as standalone conditions. The observed increase in intracellular ROS was paired with a substantial decrease of reduced glutathione (GSH; see Figure 4C) and increased oxidated glutathione (GSSG; see Figure 4D). This effect is noticeable in Figure 4E, showing the ratio of GSH/GSSG. Interestingly, TS‐TBI co‐stimulation produced a considerably higher amount of GSSG than either TS or TBI alone (Figure 4D).

### 3.4 TS exposure and TBI down‐regulates NRF2 and its detoxifying effector molecules HO‐1 and NQO‑1

**FIGURE 5:**
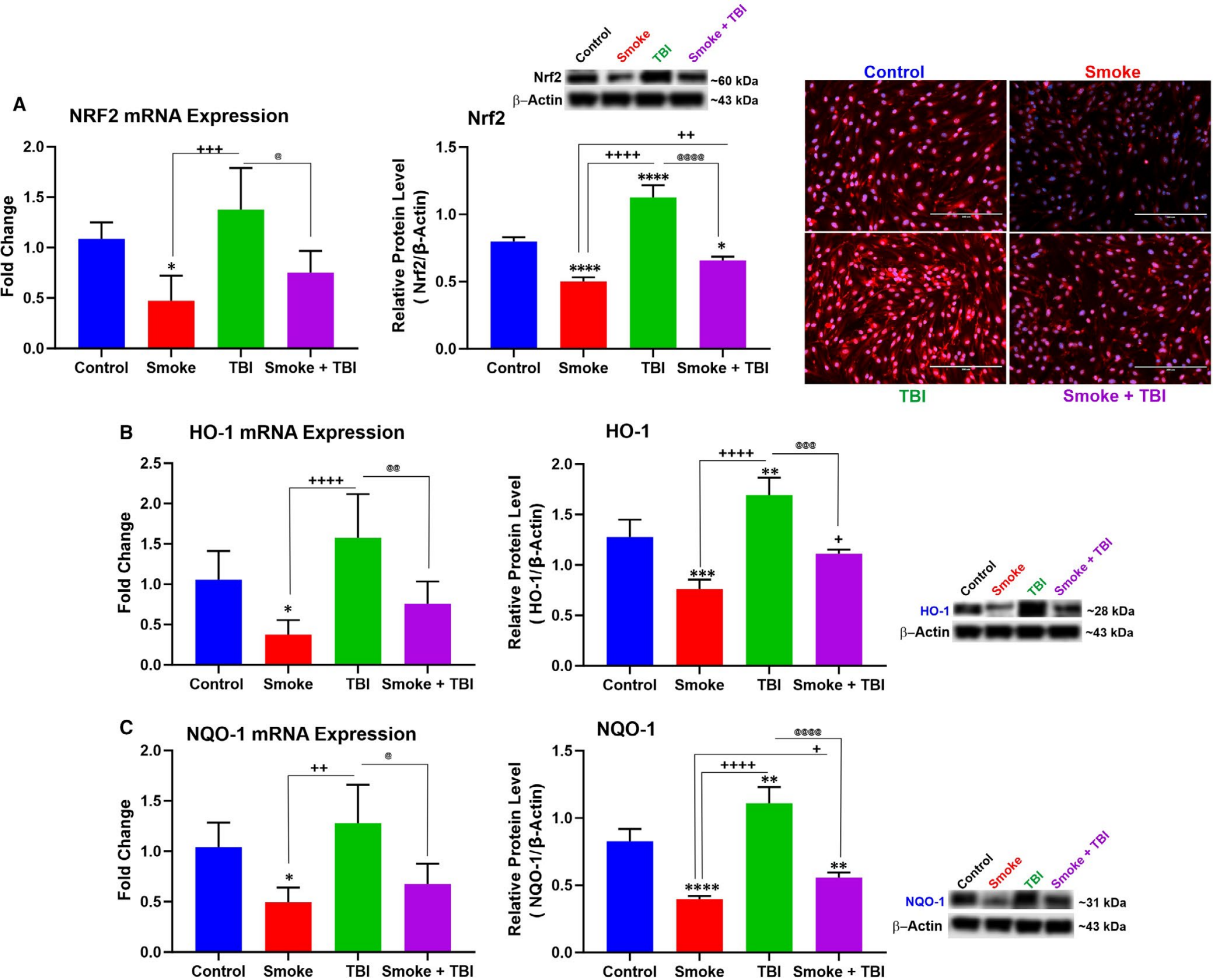
TS exposure down‐regulated Nrf2 and its downstream detoxifying effector molecules HO‐1 and NQO‐1 in BBB endothelial cells following traumatic injury simulation. A, Western blotting, quantitative RT‐PCR and (B) immunofluorescence imaging analyses emphasized the role of chronic TS exposure in Nrf2 expression level and synergism with TBI. Alternations in Nrf2 expression levels were paralleled by corresponding changes of (B) HO‐1 and (C) NQO‐1. Please note that the reported β‐actin band for Nrf2 (Figure 5A) is the same as that shown for OH‐1 (Figure 5B) since these proteins were run in the same group (Group 1). Similarly, the reported β‐actin band for NQO‐1 (Figure 5C) is the same as that shown for VECAM‐1 (Figure 3B) and Ocln (Figure 6C) since these proteins were also run in the same group (Group 3) as stated in the method section. n = 6 biological replicates, *P < .05, **P < .01, ***P < .001, ****P < .0001 vs control. ^+^ *P < *.05, ^++^ *P < *.01, ^+++^ *P < *.001, ^++++^ *P < *.0001 vs smoked group. ^@^ *P < *.05, ^@@^ *P < *.01, ^@@@^ *P < *.001, ^@@@@^ *P < *.0001 vs TBI‐induced group. WB analyses express protein/β‐actin ratios

The impact of TS exposure and TBI on Nrf2 expression was also investigated through a set of WB, IF and quantitative RT‐PCR analyses (see Figure 5). Down‐regulation of Nrf2 (protein and mRNA expression), as well as its immediate downstream detoxifying effector (see Figure 5B,C), was indicative of impairment of the antioxidative response system (ARS) caused by chronic TS exposure. Specifically, TS down‐regulated both haem oxygenase‐1 (HO‐1; Figure 5B) and NAD(P)H dehydrogenase [quinone] 1 (NQO‐1; Figure 5C). The effect was strong enough to counteract the cellular response to TBI, which produced a substantial up‐regulation of Nrf2, but this response was significantly abrogated in cells previously exposed to TS.

### 3.5 Chronic TS exposure negatively impacted BBB integrity in TBI

**FIGURE 6:**
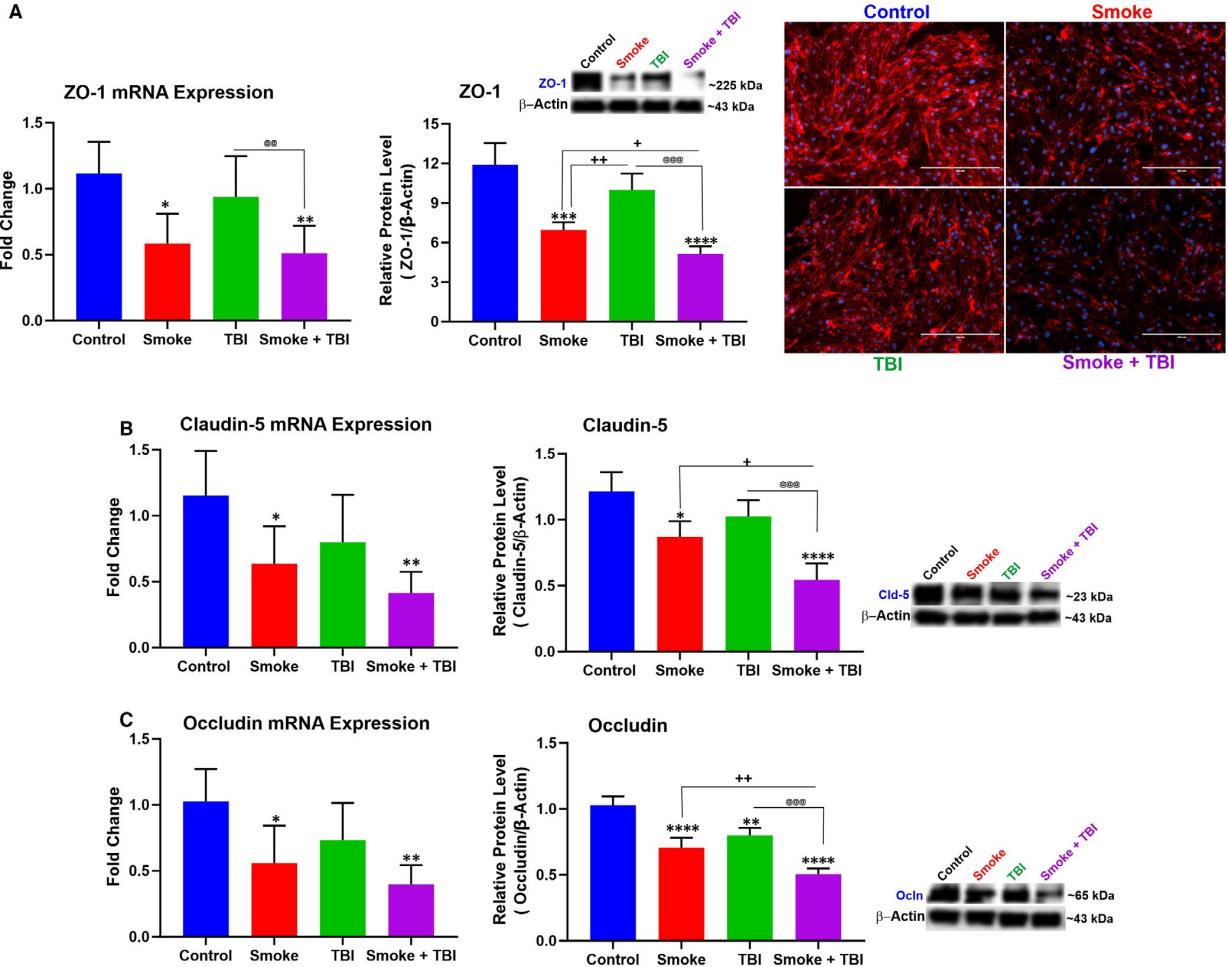
TS exposure and TBI affected cytoplasmic accessory protein ZO‐1 and TJ proteins occludin and claudin‐5. A, Western blotting, quantitative RT‐PCR and immunofluorescence imaging analyses demonstrating down‐regulation of ZO‐1 and its mRNA in cells exposed to TS and/or TBI. Data from WB and quantitative RT‐PCR analyses demonstrated a similar down‐regulation of (B) claudin‐5 and (C) occludin in cells exposed to TS and/or TBI. TBI as a standalone factor has a marginal effect compared to TS alone, thus suggests the responsibility of TS for TJ down‐regulation observed in TS‐exposed cells subjected to TBI. Please note that the reported β‐actin band for Ocln (Figure 6C) is the same as that shown for NQO‐1 (Figure 5C) and VECAM‐1 (Figure 3B) since these proteins were run in the same group (Group 3) as stated in the method section. n = 6 biological replicates, *P < .05, **P < .01, ***P < .001, ****P < .0001 vs control. ^+^ *P < *.05, ^++^ *P < *.01, ^+++^ *P < *.001, ^++++^ *P < *.0001 vs smoked group. ^@^ *P < *.05, ^@@^ *P < *.01, ^@@@^ *P < *.001, ^@@@@^ *P < *.0001 vs TBI‐induced group. WB analyses express protein/β‐actin ratios

To assess TBI and TS exposure effect on BBB integrity, we performed IF, WB and quantitative RT‐PCR analyses. Notably, we determine the expression level of zonula occludens‐1 (ZO‐1, a major BBB accessory protein) and TJ proteins primarily responsible for the low paracellular permeability of the BBB, such as occludin and claudin‐5. Both the protein expression levels and the respective mRNAs were assessed. Exposure of mBMEC cells to TS down‐regulated ZO‐1 expression and its mRNA compared to controls (Figure 6A). A similar trend was observed for claudin‐5 (Figure 6B) and occludin (Figure 6C). Both protein levels and the respective mRNAs were considerably down‐regulated compared to controls. Although the TJ protein expression was also slightly decreased by traumatic injury, our previous work supports the notion that TS acts as the main effector of the down‐regulation.^21^, ^22^, ^25^

## 4 DISCUSSION

Traumatic brain injury is among the most frequent causes of death and disability for young people. Statistically, half of the global society will experience TBI through their lifetime.^32^ Oxidative stress resulting from unbalanced ROS generation has been recognized as an essential factor in post‐traumatic secondary brain injury.^33^ TS exposure is believed to act as a prodromal factor for the onset of degenerative, neurovascular and neuroinflammatory diseases by promoting the deterioration of the ARS activity and dysfunction of the microvascular endothelial physiology.^19^, ^22^, ^31^ However, the effect of cigarette smoking as a premorbid factor on post‐TBI secondary injury and recovery is still controversial.^3^ The present work outlined the possible impact of chronic TS on the influence of TBI on brain microvascular endothelium.

Nuclear factor 2‐related is generally accepted as a critical regulator of several protective responses that control the cellular redox status in response to detrimental stress.^34^, ^35^ Usually, Nrf2 is confined in the cytoplasm by an Nrf2 inhibitor known as Kelch‐like ECH‐associated protein 1 (Keap1). Nrf2 is then ubiquitinated and processed for degradation. However, in response to oxidative/inflammatory stress conditions (endogenous and/or xenobiotics), it dissociates from Keap1 and translocates into the nucleus, where it binds to the antioxidant response element (ARE). Nrf2 binding to the ARE stimulates the transcription of more than 500 genes. These genes include regulators of redox metabolism, phase 1 and 2 enzymes, antioxidative agents (such as NADH and glutathione), promoters of ATP production and BBB TJs.^30^, ^36^ Promoting anti‐inflammatory mediators, the proteasome's activity and other transcription factors modulating the mitochondrial biogenesis are among other remarkable features of Nrf2.^37^

Recent studies have shown that the down‐regulation of Nrf2 activity and Nrf2–ARE pathway impairments aggravate the oxidative damage caused by TBI and post‐traumatic neurological injuries. These findings crucially emphasize the prominent neuroprotective role of Nrf2 in TBI and other cerebrovascular disorders.^38^, ^39^ Increased Nrf2 activity helps to reduce the burden of TBI and improve TBI outcomes by protecting BBB integrity and reducing oxidative stress and inflammation.^2^, ^12^, ^21^, ^31^, ^40^, ^41^ According to these findings, the impact of premorbid TS exposure and TBI on Nrf2 and its effector molecules NQO‐1 and HO‐1 were evaluated. Our in vitro data demonstrate that traumatic injury activates the Nrf2‐ARE pathway at the BBB endothelium (see Figure 5). These data are consistent with previous studies, confirming that TBI promotes Nrf2 expression and activity along with its downstream effectors NQO‐1 and HO‐1^5^, ^12^ while chronic TS exposure has the opposite effect.^21^, ^31^ This result further strengthens the notion of the beneficial role of Nrf2 in preventing TBI exacerbation by TS. Our data also suggest that Nrf2 may provide a promising path towards developing more efficient therapeutic strategies against TBI.

The sources of OS after TBI are cellular and molecular pathways activated in various cell types, including endothelial cells, microglia and astrocytes.^6^, ^42^ The cumulative effect of ROS following TBI seems to promote brain damage, leading to neuron loss, propagation of inflammation, increased cerebral blood flow and a loss of autoregulatory function.^43^ Measurements of intracellular ROS production and levels of GSH and GSSG confirmed the relation between oxidative stress and TBI (see Figure 4). The data revealed that TS exposure is responsible for a statistically significant increase of intracellular ROS production (see Figure 4B), as well as a substantial decline in GSH levels (see Figure 4C). Besides, the combined effect of TS exposure with traumatic injury leads to a much more sizable rise of intracellular ROS, coupled with a significant fall of reduced glutathione (GSH) and a surge of its oxidated form (GSSG) (see Figure 4E). The GSH level in TBI does not decline significantly compared to controls (see Figure 4C), which might be caused by the TBI‐responsive up‐regulation of Nrf2 since GSH production depends on the Nrf2 activity promoting glutathione synthesis and antioxidative effects.^36^

Taken together, TS exposure, as a comorbid stimulus combined with traumatic injury, abolishes the post‐traumatic activation/up‐regulation of Nrf2 and prevents this physiological recovery system's activation. Nrf2 down‐regulation is responsible for further impairment of the BBB. Recent studies have shown BBB impairment as an essential element of secondary brain injury affecting TBI outcomes.^44^ As demonstrated by our data, TS substantially down‐regulates the expression levels of occludin and claudin‐5. These TJ proteins are considered primarily responsible for maintaining the low paracellular permeability of the BBB. Thus, down‐regulation of their expression levels can significantly impact the integrity of the endothelial barrier.

Furthermore, these TJs are anchored to the cell cytoskeleton through ZO‐1, which helps direct their distribution around the cell membrane.^45^ Hence, a down‐regulation of ZO‐1 can affect these TJs' cellular distribution, further compromising the integrity of the vascular endothelial layer of the BBB. BBB integrity is remarkably affected by OS. Unbalanced ROS production can alter TJ protein expression, promote endothelium dysfunction and lose BBB integrity.^46^, ^47^

Moreover, inflammation has been linked to oxidative stress caused by TS through sequential events leading to NF‐κB activation and the up‐regulation of inflammatory factors, including vascular adhesion molecules and cytokines.^48^, ^49^ Sustained and excessive inflammation promoted by inflammatory mediators penetrating through the leaky BBB may interfere with the brain's normal restorative processes and promote subsequent neurological impairment.^11^, ^50^ On the other hand, inflammatory molecules can further impair the BBB's integrity by promoting the loss of occludin/ZO‐1 and other TJ proteins.^15^, ^51^ Thus, it is unsurprising that OS and inflammation have been widely recognized as essential negative contributors to the TBI pathophysiology and post‐TBI neuronal damage.^33^, ^52^ In this respect, TS prompted the inflammatory transcription factor NF‐kB overexpression along with inflammatory cytokines and vascular adhesion molecules (see Figure 2) while down‐regulating the TJ proteins (see Figure 6). These data well cope with the notion that TS‐induced OS and inflammation will likely worsen the loss of vascular integrity and TBI outcome.^33^, ^53^

Nrf2 reduces the OS imbalance and consequently negatively modulates the redox‐sensitive NF‐κB signalling pathway that promotes neuroinflammation.^54^ Consistent with the Nrf2‐NF‐κB interplay, pro‐apoptotic mediators are down‐regulated by the cytoprotective activity of Nrf2 while are up‐regulated by NF‐κB.^55^, ^56^ Other studies have shown enhanced NF‐κB activation and the up‐regulation of pro‐inflammatory cytokines in the brain and spinal cord injury in Nrf2^‐/‐^ mice compared to their wild‐type Nrf2^+/+^ counterparts.^57^ As we show in the results, the downstream effect of NF‐kB up‐regulation and activation is reflected in the increased expression of various pro‐inflammatory markers we assessed in this study. These include multiple cytokines as well as vascular endothelial adhesion molecules (see Figures 2 and 3). Also, there is an inverse correspondence between the effect of metformin and rosiglitazone on Nrf2 levels and the resulting effect on Nf‐KB expression since these two nuclear factors negatively regulate each other.^58^ These data indicate a higher severity of post‐traumatic endothelial injury in premorbid TS consistent with the mechanisms boosting TBI's secondary brain injury.^53^, ^59^

An additional risk factor for TBI patients is the effect of TS on blood haemostasis regulation. In response to vessel damage and/or inflammation, thrombomodulin (a key element of the anticoagulant protein C pathway) released by the endothelium blocks the prothrombinase complex's activity, thus arresting the undesired coagulation cascade.^60^ Therefore, a decreased release of this anticoagulant factor would inherently increase the risk for the coagulation cascade to run unchecked. Unwanted blood coagulation could further restrict local blood flow and increase the damage of the affected vascular district (and the surrounding undamaged areas). TS exposure has been shown to boost down‐regulation of thrombomodulin.^21^, ^31^ Although reducing the risk of cerebral haemorrhage could benefit TBI patients, the decreased ability to control the blood coagulation cascade increases the risk for unwanted blood clot formation and stroke.^31^

There are study limitations that we need to acknowledge. The present work does not address the underlying mechanisms of how TS exposure may down‐regulate Nrf2 expression. This mechanism of action is currently not well understood. However, of relevant importance is the fact that cigarette smoke is highly enriched with ROS, RNS, NO and free radicals of organic compounds,^61^ besides other stable substances that have the potential to increase ROS production^62^ and interact with enzymes that are responsible for ROS generation, such as Nox oxidases.^63^ Previous studies have shown that cigarette smoke extracts increased the expression of Nox2 (expressed in various brain regions), thus promoting the activation of the Nox2‐NF‐kB system leading to the repression of the Nrf2 pathway since NF‐kB and Nrf2 regulate each other negatively.^58^ This mechanistic hypothesis that needs to be fully validated is indirectly supported by the fact that previous studies by us and others have found that the antidiabetic drug metformin prevents Nrf2 down‐regulation by chronic TS exposure ^31^, ^64^ where AMPK activation by metformin effectively prevents Nox2 activation.^65^ However, other pathway‐specific modulators/promoters may be implicated as well.^21^ As shown by previous studies, rosiglitazone, another antidiabetic drug in the thiazolidinedione class that has been shown to counteract TS‐induced down‐regulation of Nrf2,^66^ is a peroxisome proliferator‐activated receptor‐gamma (PPAR‐gamma) agonist. PPAR‐γ agonists have been shown to suppress inflammation consistently via NF‐κB inhibition.^67^

The mechanistic component underlying the relationship between ROS and Nrf2 down‐regulation by TS exposure was beyond the scope of this more observational study. However, in upcoming studies, we plan to dissect these still nebulous underlying mechanisms through which TS may promote Nrf2 down‐regulation and affect TBI outcome. We also plan to evaluate the effectiveness of additional Nrf2 modulators to help reduce the burden of TBI. Overall, the development of timed interventions aimed at interfering with different aspects of the oxidative and inflammatory response systems would likely help developing new approaches to reduce the burden of secondary post‐traumatic injuries.

## 5 CONCLUSION

Chronic TS is among the preeminent causes of preventable mortality in the United States.^68^ Yearly, TS has affected the lives of about six million people globally, and more than half a million people in the United States are killed by TS.^4^, ^14^ Smoking increases the onset and progression of major neuroinflammatory and cerebrovascular diseases through excitotoxicity, OS, cerebral oedema formation, neuroinflammation and impairment of BBB endothelial physiology. In this work, we assessed the effect of TS on TBI‐induced damage of the BBB endothelium using an in vitro traumatic injury cell culture model based on mBMEC cells. Our data suggest that TS promotes the exacerbation of brain microvascular endothelial damage caused by traumatic injuries. The detrimental effects of TS are driven by the impairment of the ARS primarily controlled through Nrf2. These effects manifest as increased cellular oxidative stress, inflammation and down‐regulation of TJs, leading to BBB integrity loss. Our data provide further evidence verifying the damaging role of premorbid TS in exacerbating post‐TBI injuries.

## CONFLICT OF INTEREST

The authors declare no conflict of interests.

## AUTHOR CONTRIBUTION

**Farzane Sivandzade:** Conceptualization (lead); Data curation (equal); Writing‐original draft (lead). **Faleh Alqahtani:** Conceptualization (equal); Data curation (equal); Writing‐original draft (supporting). **Luca Cucullo:** Conceptualization (equal); Data curation (equal); Funding acquisition (lead); Resources (lead); Supervision (lead); Writing‐review & editing (lead).

## Data Availability

The data that support the findings of this study are available from the corresponding author upon reasonable request.
